# Supplementary material for: Cell-TRACTR: A transformer-based model for end-to-end segmentation and tracking of cells
Source: PLoS Comput Biol. 2025 May 23;21(5):e1013071. doi: 10.1371/journal.pcbi.1013071 (PMC12101859; doi:10.1371/journal.pcbi.1013071)
Supplement: S1 Text — (PDF) [file pcbi.1013071.s001.pdf]

Supplementary Text

Cell Tracking Challenge metric (OP_{CTB}) and limitations for frequently-dividing cells

The Cell Tracking Challenge metric for quantifying overall performance of the cell tracking benchmark, OP_{CTB} (Equation S1), incorporates measures of both tracking accuracy (TRA) and segmentation accuracy (SEG).

$$OP_{CTB} = 0.5 * (TRA + SEG) \ (S1)$$

Acyclic oriented graphs are employed to compute the TRA score. The spatial locations of cells are denoted by vertices while the edges signify the temporal associations (1). For each series of time-lapse images, an acyclic oriented graph is generated based on the output from a tracking algorithm. These graphs are then compared to a reference graph which contains the information from the ground truth. The acyclic oriented graph matching (AOGM) measure is a weighted sum of the number of operations needed to transform the predicted graph into the reference graph (Equation S2). These operations include *split vertex* (NS), *add vertex* (FN), *delete vertex* (FP), *delete edge* (ED), *add edge* (EA), *alter the edge semantics* (EC), where we follow the notation used by the Cell Tracking Challenge. The AOGM is a weighted sum of the number of the operations required to match the two graphs.

$$AOGM = w_{NS}NS + w_{FN}FN + w_{FP}FP + w_{ED}ED + w_{EA}EA + w_{EC}EC \ (S2)$$

where $w_{NS} = 5$, $w_{FN} = 10$, $w_{FP} = 1$, $w_{ED} = 1$, $w_{EA} = 1.5$, and $w_{EC} = 1$. $AOGM_0$ is the cost needed to generate the reference graph from scratch. AOGM is normalized to $AOGM_0$ and subtracted from 1 to get the TRA score (Equation S3). To ensure the score remains positive, the AGOM score cannot be greater than the $AOGM_0$ score. If tracking is perfect, resulting in AGOM = 0, then the tracking score TRA = 1.

$$TRA = 1 - \frac{\min(AOGM, AOGM_0)}{AOGM_0} \ (S3)$$

As in the main text, we define a 'tracker cell' as a cell predicted by the tracking algorithm and a 'ground truth cell' as a cell defined in the ground truth. In the case of perfect tracking, each tracker cell would be uniquely affiliated with one ground truth cell, however errors in tracking cause deviations from this. In the OP_{CTB} metric, a tracker cell needs to overlap with at least half the area of a ground truth cell to be considered a match. A tracker cell without a match is a false positive and a ground truth cell without a match is a false negative. A tracker cell can match with multiple ground truth cells, but a ground truth cell can only match with at most one tracker cell. The *split vertex* operation is used when a tracker cell matches with multiple ground truth cells whereas the *add vertex* and *delete vertex* operations are used for false negatives and false positives, respectively. The operations *add edge* and *delete edge* are used to add and remove temporal connections between cells. Lasty, the *alter the edge semantics* operation modifies existing temporal connections.

The Cell Tracking Challenge uses the SEG score to measure localization accuracy. The Jaccard Similarity index is used to calculate the intersection over union (IOU) between each ground truth cell (GT) and matched tracker cell (TrCell) (Equation S4).

$$J(\text{TrCell}, GT) = \frac{|GT \cap \text{TrCell}|}{|GT \cup \text{TrCell}|} \quad (S4)$$
$$where \quad |GT \cap \text{TrCell}| > 0.5 * |GT|$$

The SEG score is the Jaccard Similarity index averaged over all the ground truth cells (Equation S5).

$$SEG = \frac{1}{N_{GT}} \sum_{1}^{N_{GT}} J(\text{TrCell}, GT) \quad (S5)$$

There are some drawbacks to using OP_{CTB}, especially in cases where cell division events are frequent, and the timing of division is not precisely known. In some cases, removing division links, thus breaking cell lineages that should remain intact, can increase the TRA score despite being a worse tracking prediction. As a reminder, a higher TRA score is better than a lower TRA score. For example, a division event predicted a frame early or late relative to the ground truth can result in a lower TRA score than the equivalent case with division entirely removed (Fig S4). This decrease in score occurs because the predicted division creates edges that need to be removed to recreate the reference graph. When examining Cell-TRACTR's performance on a movie from the test set, 5 of 5 *split vertex* operations and 20 out of 21 *add edges* operations occurred when a cell division was predicted late by one frame (Movie S3). Thus, a significant portion of the error used to calculate the TRA score comes from early and late divisions which is undesirable for evaluating tracking performance since these are somewhat arbitrary distinctions because it is challenging to generate ground truths that pinpoint the exact frame a cell divides. As a result, test sets used to evaluate the cell tracking algorithms are bound to contain ambiguous cell division events. The TRA score will measure the algorithm's ability to exactly match imperfect ground truths instead of measuring the algorithm's ability to track cell divisions at a broader level.

OP_{CTB} versus Cell-HOTA

Cell-HOTA offers several potential benefits over the OP_{CTB} score. With OP_{CTB}, the TRA and SEG scores are completely independent of each other. The TRA score serves as the detection and tracking accuracy whereas the SEG score provides the localization accuracy. The TRA score is reported at one specific similarity threshold α. Therefore, it is hard to gauge how the tracking algorithm will perform with a stricter or looser similarity threshold. Although the SEG score provides information about the localization accuracy, it is difficult to relate this to the TRA score. In contrast, the Cell-HOTA score integrates the localization accuracy into DetA, AssA, and DivA by evaluating them at multiple similarity thresholds.

Another issue with the OP_{CTB} is that the TRA score lacks interpretability. The TRA score uses detection operations (*add vertex*, *remove vertex*, *split vertex*) that are comprehensible, however the tracking operations (*add edge*, *remove edge*, *alter the edge semantics*) do not clearly translate into understandable errors. In addition, the *split vertex* operation is used to correct late divisions however there is no inverse relationship. Plus, it is difficult to specifically evaluate division accuracy, which is critical for cell tracking. Conversely, Cell-HOTA breaks down into DetA, AssA, and DivA which facilitates straightforward evaluation of the algorithm's capacity to track cells. For example, if a small percentage of cells in a movie are detected but they are all tracked correctly, then DetA would be low and AssA would be high. However, if most cells in a movie are detected but they are tracked incorrectly, then DetA would be high and AssA would be low. Interpretability of these scores is crucial for figuring out how to improve an algorithm.

Lastly, the OP_{CTB} is weighted heavily towards localization accuracy, as the score is computed as the average of the SEG and TRA scores, where SEG represents the localization accuracy and TRA represents the detection and association accuracy. Ideally, a tracking metric should balance the detection, association, and localization accuracy. The Cell-HOTA score effectively addresses this by weighing the DetA and AssDivA equally and incorporating localization accuracy into both of those metrics.

Directly comparing Cell-HOTA and OP_{CTB} results

In order to directly compare Cell-HOTA with TRA from OP_{CTB}, we need to look at the Cell-$HOTA_{0.5}$ score. Cell-$HOTA_{0.5}$ measures the overall detection and tracking accuracy at $\alpha = 0.5$. Therefore, a tracker cell and ground truth cell only match if the IOU is greater than 0.5. Similarly, the TRA score measures the overall

detection and tracking accuracy where a tracker cell needs to overlap with at least half the area of the ground truth cell to be considered a match. When assessing model performance on the mammalian DeepCell dataset, Cell-TRACTR achieves a high Cell-$HOTA_{0.5}$ score, but has a comparatively lower TRA score. Even when ignoring $DivA_{0.5}$, a condition which would most closely resemble the TRA score since it puts little weight on cell division, Cell-TRACTR still attains comparatively high $DetA_{0.5}$ and $AssA_{0.5}$ scores. This discrepancy is due to how the TRA score accounts for false positives and false negatives. The Cell-HOTA metric gives equal weight to false negatives and false positives whereas the TRA score gives ten times the weight to false negatives compared to false positives. The weight configuration for the AGOM measure used to generate the TRA score was designed to reflect the effort needed to correct an error manually (1). When analyzing the TRA score on the mammalian DeepCell dataset, EmbedTrack accrued 4080 false positives and 150 false negatives whereas Cell-TRACTR had 1374 false positives and 692 false negatives. Although Cell-TRACTR had significantly fewer false positives and false negatives combined, the high weight towards false negatives had a more significant impact on the TRA score.

Supplementary Methods

Query selection

To give object queries more context, query selection generates region proposals from the encoder output (2) (Fig S1). Each pixel in the multi-scale features acts as a potential region proposal, predicting a bounding box, segmentation mask, and class label. The top-K scoring region proposals are selected to serve as object queries for the decoder. For region proposals that are classified as "cell", there is a strong correlation between the pixel location of the regional proposal within the multi-scale features and the predicted location of the cell in the final layer of the decoder (Fig S8). In general, we found that Cell-TRACTR preferentially used the low-resolution multi-scale features for the bacterial mother machine dataset (Fig S9) and high-resolution multi-scale features for the mammalian DeepCell dataset (Fig S10). Following Mask-DINO (3), segmentation masks are used to generate the positional embeddings for the object queries. To minimize computational load, backpropagation is performed only for the top-K scoring region proposals.

Adding noise to bounding boxes during training

Random noise (Δx, Δy) is generated by selecting two numbers from a uniform distribution ranging between -1 to 1. We use noise coefficients λ_1 and λ_2, with default values of 0.2 and 0.1 respectively, for center shifting and box scaling respectively. These noise coefficients scale the amount of random noise added to the boxes. Center shifting affects the (x, y) coordinates of the box, whereas box scaling affects the height and width of the box.

Matching algorithm

During training, we use the Hungarian algorithm to match each ground truth to a prediction (4). For object detection, each ground truth cell is optimally matched to a predicted cell using this approach. This optimization is based on the class label, bounding box, and segmentation mask similarities. When cells are being tracked, the predicted cells are automatically matched with their respective ground truths. The remaining ground truth cells which are not tracked, like cells entering the field of view, are matched to a predicted cell derived from an object query. For cell division, a set of two predicted cells need to match with a set of two ground truth cells. However, this could be problematic as the daughter cells are matched as a set, not as individual cells. The order of the daughter cells should match the order of the ground truth cells. Predicted daughters could be matched to the ground truth daughters of a reversed order. To address the ambiguity of the order in cell division, we use the Hungarian algorithm to pick the correct order of ground truth cells.

Reference points for cell divisions

Most DETR-like models that track objects do not deal with dividing objects (5–9). Therefore, in classical DETR-like models, each query will only predict one class label, bounding box, and segmentation mask. Below, we describe the approach that is used in DETR-like models which we also employ in Cell-TRACTR; in the following paragraph we discuss the extensions we made to handle cell division that are specific to Cell-TRACTR. In the decoder, the bounding boxes are converted into positional embeddings to assist in self-attention and cross-attention. However, the raw bounding boxes are also used as reference points for cross-attention. Queries attend to a small set of sampling points around the reference point (2). Instead of each query attending to the whole feature map, it will attend to a specific area on the feature map. Deformable attention reduces time to convergence and computational cost. Since deformable attention was designed for image processing, it is not used during self-attention. For each layer in the decoder, iterative bounding box refinement is performed where the decoder refines the bounding box prediction. These bounding boxes are then used to generate the reference points and positional embeddings needed for the next layer.

In Cell-TRACTR, there are two predictions made per query (Fig S11). For object queries, only the first prediction is utilized. However, track queries may predict a cell division. For track queries that predict a cell division, the combined bounding box between the two divided cells is used to generate the reference point and positional embedding. Like the original method, the decoder refines the bounding box prediction for each of the divided cells separately. The combined bounding box is used to generate the reference points and positional embeddings needed for the next layer.

Alleviating the conflict between object and track queries

While simultaneously detecting and tracking cells makes DETR-based models powerful, it also has drawbacks. Various studies have highlighted that conflict between object and track queries can lead to an overall decrease in detection accuracy (6–9). MOTRv2 (6) improves on MOTR (10) by utilizing the YOLOX (11) object detector to provide the positional embeddings for the object queries so the model can focus on association. MOTRv3 (7), which builds on top of MOTR (10), employs a Release-Fetch Supervision strategy that utilizes one-to-one matching between all queries and all objects in the first five layers and then one-to-one matching only between the object queries and newborn objects in the last layer. MeMOTR (8) simply uses the first decoder layer for object detection only and uses the subsequent decoder layers for joint detection and tracking. Similar to MOTRv3, CoMOT (9) implements Coopetition Label Assignment where the first five decoder layers perform an additional one-to-one matching with just the object queries and all objects (newborn and tracked) and then a standard last layer. Following these advances, we adopt the first decoder layer as a detection only layer like MeMOTR (Fig S5) and add an extra one-to-one matching between object queries and all objects in the subsequent layers except for the last layer, similar to the approach used in CoMOT (Fig S2).

Track Group Denoising (TGD) and Query Denoising (QD)

For faster convergence during training, we added denoised track group queries and denoised track queries as proposed in MotrV3 (7) and MotrV2 (6). Both training methods consist of the model learning to track noisy queries. Since the model simultaneously processes the original track queries alongside both the noised track group queries and noised track queries, it is important to regulate information flow across these training techniques. For example, the original track queries could cheat by leveraging information about a denoised track query. To prevent this, we employ attention masks to selectively block the attention mechanism from accessing certain parts of the input data (12,13) (Fig S12). Specifically, the masks are added to the attention scores before the softmax operation, setting the scores for the blocked parts to a very large negative value. This ensures that these parts have a negligible effect after the softmax, effectively isolating the attention to focus only on the relevant, unmasked portions of the data.

For TGD, random noised is added to the bounding boxes predicted from the previous frame and converted into positional embeddings (Fig S12). Following Ref. (6), we use noise coefficients of $\lambda_1 = 0.2$ and $\lambda_2 = 0.1$.

Random noise, drawn from separate normal distributions with a mean of 0 and standard deviation of 0.1, is added to the content embeddings. TGD teaches the model to generalize and become less dependent on precise positional and content information. Unlike MotrV3, to reduce computational load we use only one track group, whereas they use multiple track groups. This TGD method, inspired by Group-DETR (14), has been shown to increase overall tracking performance (7).

For QD, all ground truth bounding boxes in the previous frame are collected. A small amount of noised is added to each bounding box (Fig S12). The noise coefficients, $\lambda_1 = 0.2$ and $\lambda_2 = 0.1$, are the same as used in TGD. The noised bounding boxes are then converted into positional embeddings. The same learned content embedding is used for all noised track queries. Due to the lack of semantic information, the model is forced to denoise the noised track queries solely based on the positional information. This method, which was introduced in DN-DETR (13), helps the model generalize and speed up training.

TGD and QD are very similar in implementation but differ in terms of targeting training efficiency and tracking performance. For TGD, positional embeddings originate from the predicted bounding boxes while for QD, the positional embeddings originate from the ground truth boxes. In addition, for TGD, the predicted content embeddings are utilized, whereas for QD, all noised track queries use the same learned content embedding. At a high level, TGD primarily improves tracking performance while QD primarily speeds up training. TGD and QD are only used during training. As a result, they do not add any computational load during inference.

Measuring CUDA memory usage during training

Cell-TRACTR is computationally expensive due to the image-sized segmentation masks generated for each cell. This challenge becomes more pronounced when there when the number of cells in an image is large. For the bacterial mother machine dataset, training was conducted using a V100 GPU without encountering computational constraints. This is largely because the images were relatively small (256x32 pixels) and contained a limited number of cells (up to ~14 cells).

However, when training Cell-TRACTR on the DeepCell dataset, computational challenges arose. The larger image dimensions (584x600 pixels) and higher number of cells (up to ~300 cells) per image resulted in CUDA memory requirements exceeding the capacity of a V100 GPU. To address this, we utilized an A100 GPU, which provided the necessary memory to train the model effectively.

To quantify computational costs, we measured the peak CUDA memory usage during training and compared it to the total number of queries processed by the model (Fig S13). Max CUDA memory usage is plotted against the total number of queries. Each iteration begins with object detection in the first frame, followed by tracking in the subsequent two frames. The total number of queries represents those fed into the decoder at the final frame, with a minimum of 400 object queries. Memory usage was recorded for each iteration over one training epoch.

As the number of total queries increased, the max CUDA memory usage increased. Notably, the segmentation masks were kept at the same resolution as the raw images during training. However, reducing the resolution of the segmentation masks to one-quarter of the raw image size (Fig S13B), as implemented in Mask DINO (3) and DETR (15), would significantly lower CUDA memory, though at the cost of reduced segmentation precision. Additionally, there were no computational constraints during inference for either dataset. The A100 GPU was not necessary for evaluating Cell-TRACTR on the DeepCell dataset. Inference was performed on a single V100 GPU for this analysis.

Supplementary References

1. Matula P, Maška M, Sorokin DV, Matula P, Ortiz-de-Solórzano C, Kozubek M. Cell Tracking Accuracy Measurement Based on Comparison of Acyclic Oriented Graphs. Abraham T, editor. PLOS ONE. 2015 Dec 18;10(12):e0144959.

2. Zhu X, Su W, Lu L, Li B, Wang X, Dai J. Deformable DETR: Deformable Transformers for End-to-End Object Detection. ArXiv201004159 Cs [Internet]. 2021 Mar 17 [cited 2022 Apr 13]; Available from: http://arxiv.org/abs/2010.04159

3. Li F, Zhang H, xu H, Liu S, Zhang L, Ni LM, et al. Mask DINO: Towards A Unified Transformer-based Framework for Object Detection and Segmentation [Internet]. arXiv; 2022 [cited 2022 Nov 14]. Available from: http://arxiv.org/abs/2206.02777

4. Kuhn HW. The Hungarian method for the assignment problem. Nav Res Logist Q. 1955 Mar;2(1–2):83–97.

5. Meinhardt T, Kirillov A, Leal-Taixe L, Feichtenhofer C. TrackFormer: Multi-Object Tracking with Transformers [Internet]. arXiv; 2022 [cited 2022 Nov 14]. Available from: http://arxiv.org/abs/2101.02702

6. Zhang Y, Wang T, Zhang X. MOTRv2: Bootstrapping End-to-End Multi-Object Tracking by Pretrained Object Detectors [Internet]. arXiv; 2022 [cited 2022 Nov 27]. Available from: http://arxiv.org/abs/2211.09791

7. Yu E, Wang T, Li Z, Zhang Y, Zhang X, Tao W. MOTRv3: Release-Fetch Supervision for End-to-End Multi-Object Tracking [Internet]. arXiv; 2023 [cited 2023 Jul 17]. Available from: http://arxiv.org/abs/2305.14298

8. Gao R, Wang L. MeMOTR: Long-Term Memory-Augmented Transformer for Multi-Object Tracking [Internet]. arXiv; 2023 [cited 2023 Aug 7]. Available from: http://arxiv.org/abs/2307.15700

9. Yan F, Luo W, Zhong Y, Gan Y, Ma L. Bridging the Gap Between End-to-end and Non-End-to-end Multi-Object Tracking [Internet]. arXiv; 2023 [cited 2023 Jul 10]. Available from: http://arxiv.org/abs/2305.12724

10. Zeng F, Dong B, Zhang Y, Wang T, Zhang X, Wei Y. MOTR: End-to-End Multiple-Object Tracking with Transformer. 2021 May 7;17.

11. Ge Z, Liu S, Wang F, Li Z, Sun J. YOLOX: Exceeding YOLO Series in 2021 [Internet]. arXiv; 2021 [cited 2024 May 23]. Available from: http://arxiv.org/abs/2107.08430

12. Vaswani A, Shazeer N, Parmar N, Uszkoreit J, Jones L, Gomez AN, et al. Attention is All you Need. :11.

13. Li F, Zhang H, Liu S, Guo J, Ni LM, Zhang L. DN-DETR: Accelerate DETR Training by Introducing Query DeNoising [Internet]. arXiv; 2022 [cited 2022 Nov 14]. Available from: http://arxiv.org/abs/2203.01305

14. Chen Q, Chen X, Wang J, Feng H, Han J, Ding E, et al. Group DETR: Fast DETR Training with Group-Wise One-to-Many Assignment [Internet]. arXiv; 2022 [cited 2022 Nov 14]. Available from: http://arxiv.org/abs/2207.13085

15. Carion N, Massa F, Synnaeve G, Usunier N, Kirillov A, Zagoruyko S. End-to-End Object Detection with Transformers [Internet]. arXiv; 2020 [cited 2022 Nov 14]. Available from: http://arxiv.org/abs/2005.12872
