# Supplementary figures and images for: Cell-TRACTR: A transformer-based model for end-to-end segmentation and tracking of cells
Source: PLoS Comput Biol. 2025 May 23;21(5):e1013071. doi: 10.1371/journal.pcbi.1013071 (PMC12101859; doi:10.1371/journal.pcbi.1013071)

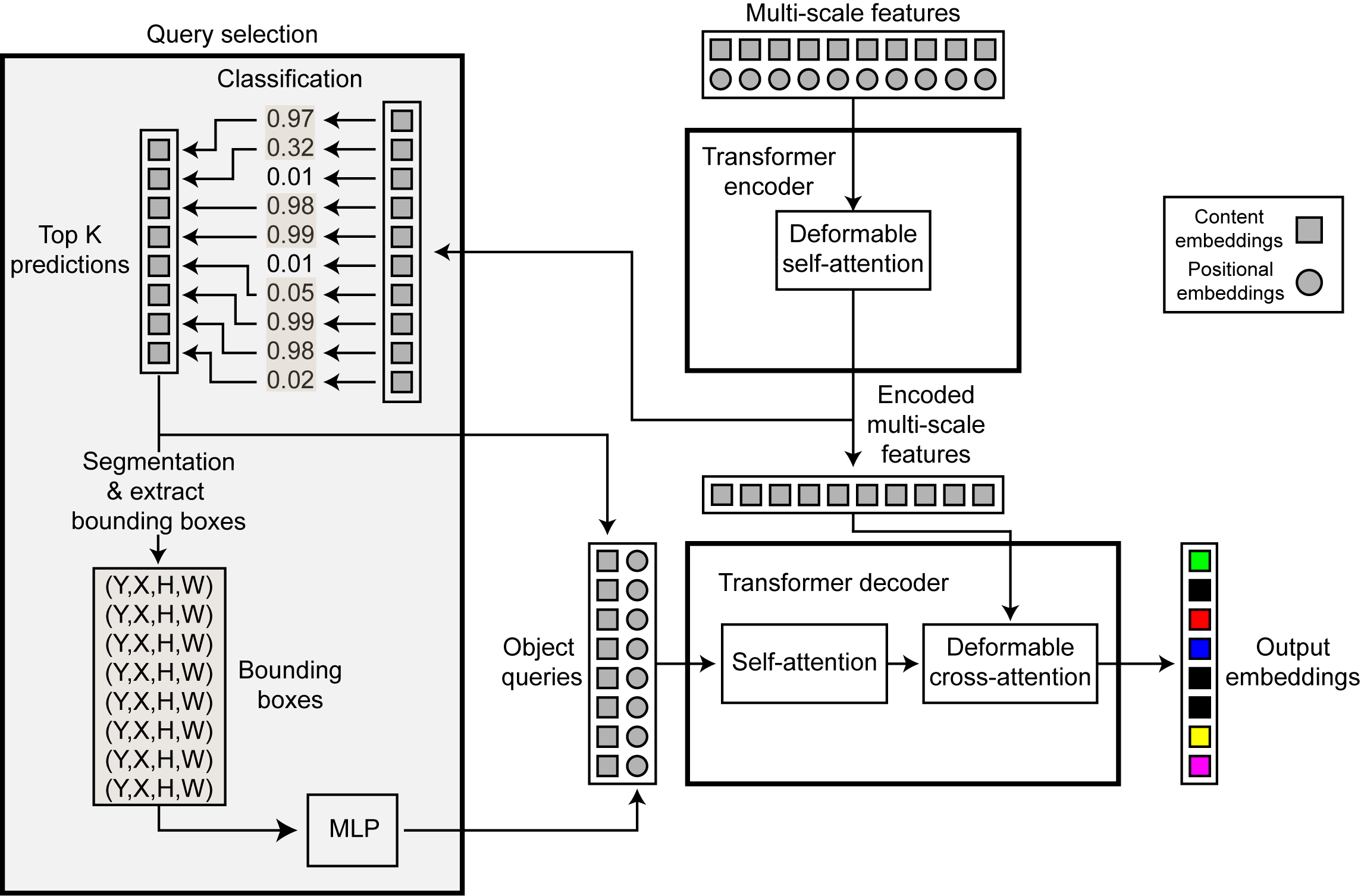

Supplement: S1 Fig — (TIF) [file pcbi.1013071.s005.tif]

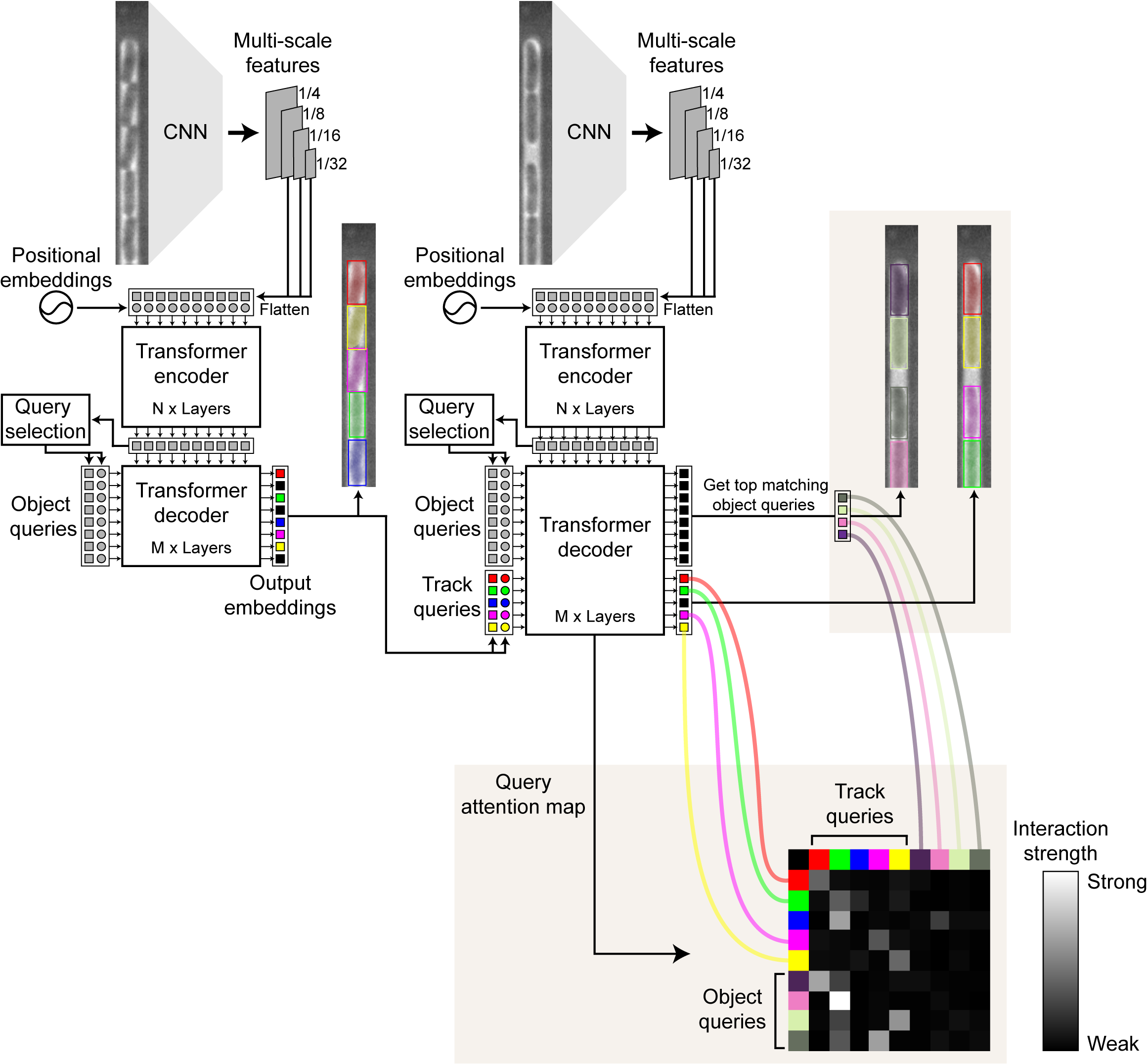

Supplement: S2 Fig — (TIF) [file pcbi.1013071.s006.tif]

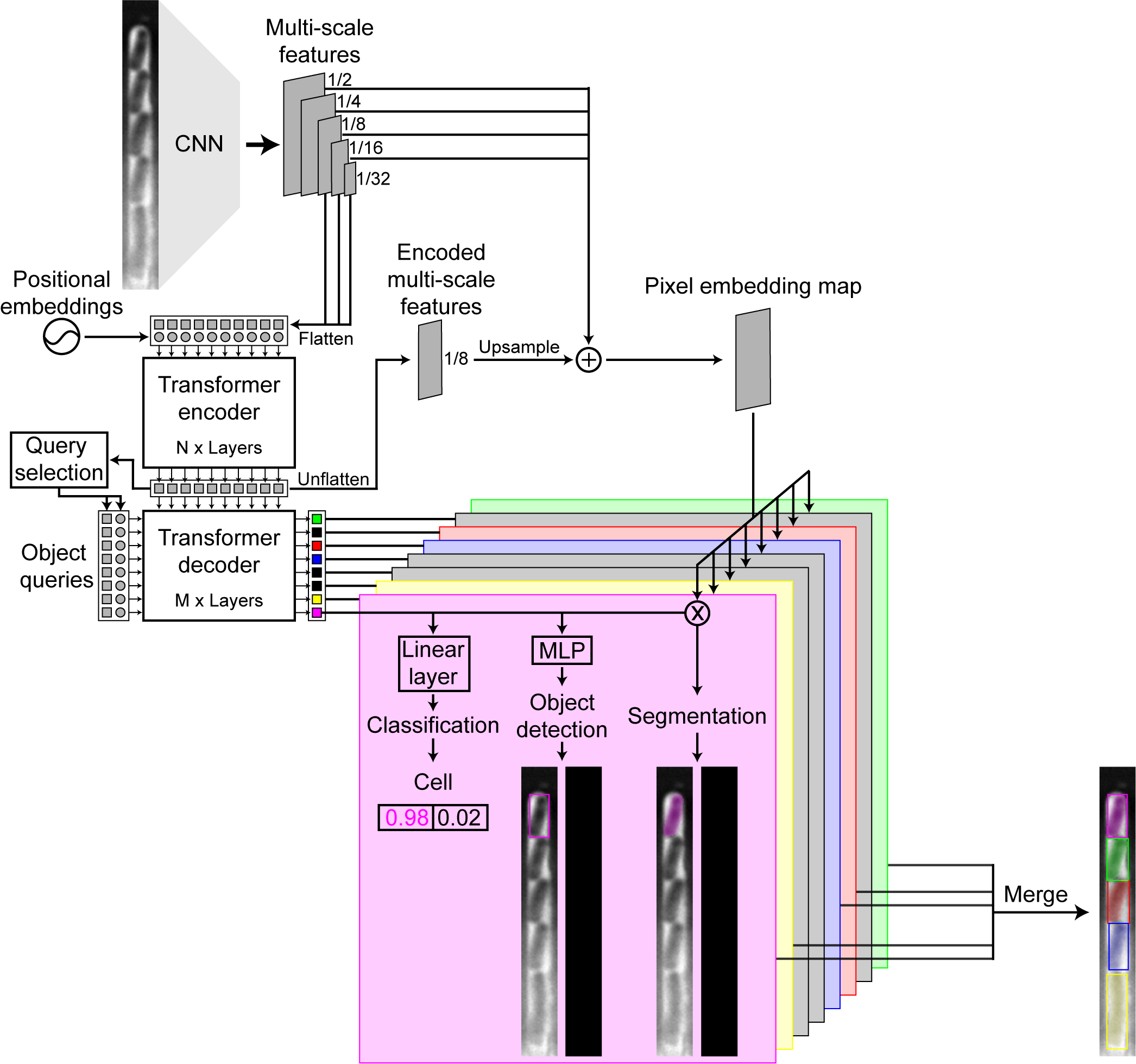

Supplement: S3 Fig — (TIF) [file pcbi.1013071.s007.tif]

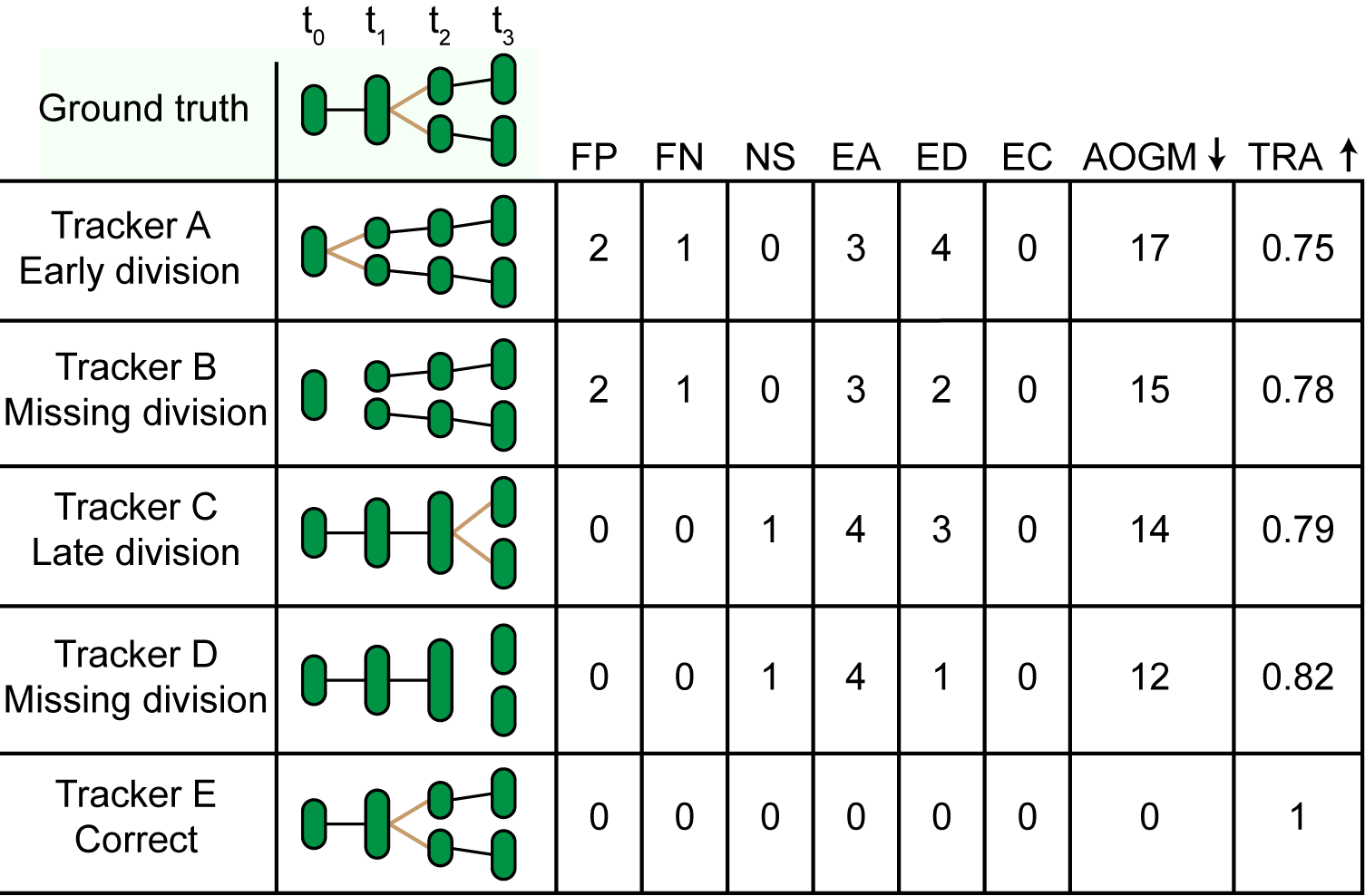

Supplement: S4 Fig — (TIF) [file pcbi.1013071.s008.tif]

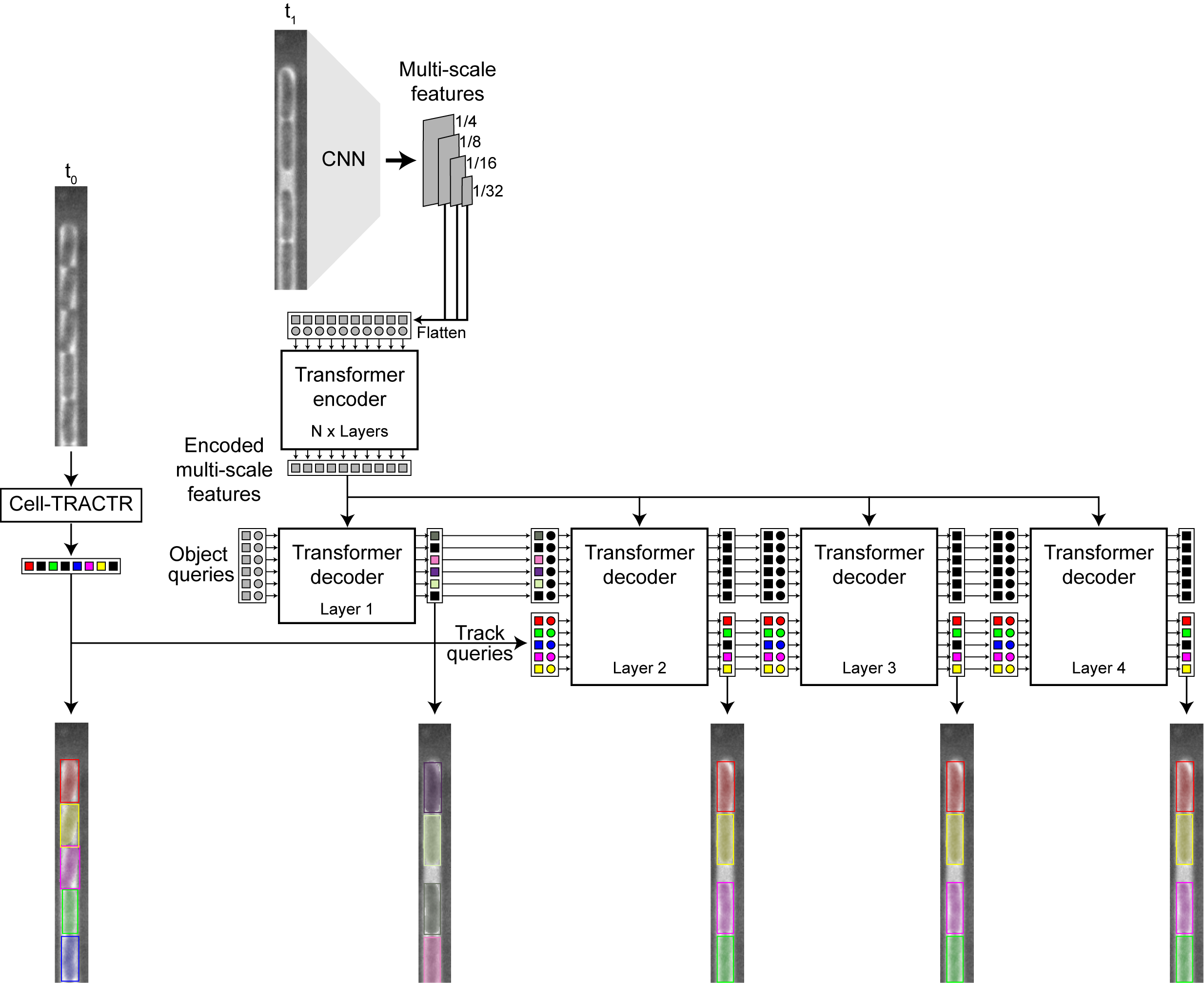

Supplement: S5 Fig — (TIF) [file pcbi.1013071.s009.tif]

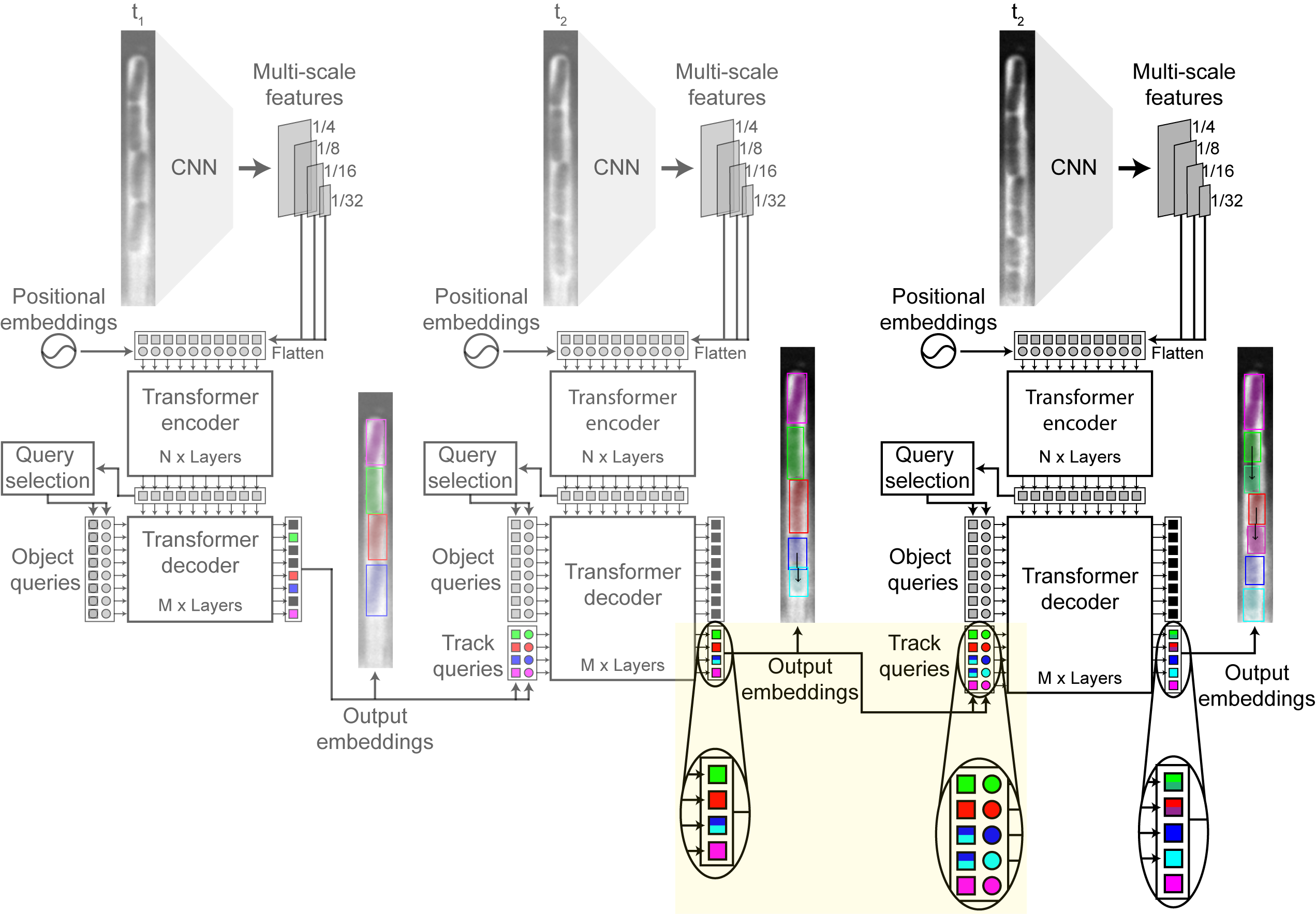

Supplement: S6 Fig — (TIF) [file pcbi.1013071.s010.tif]

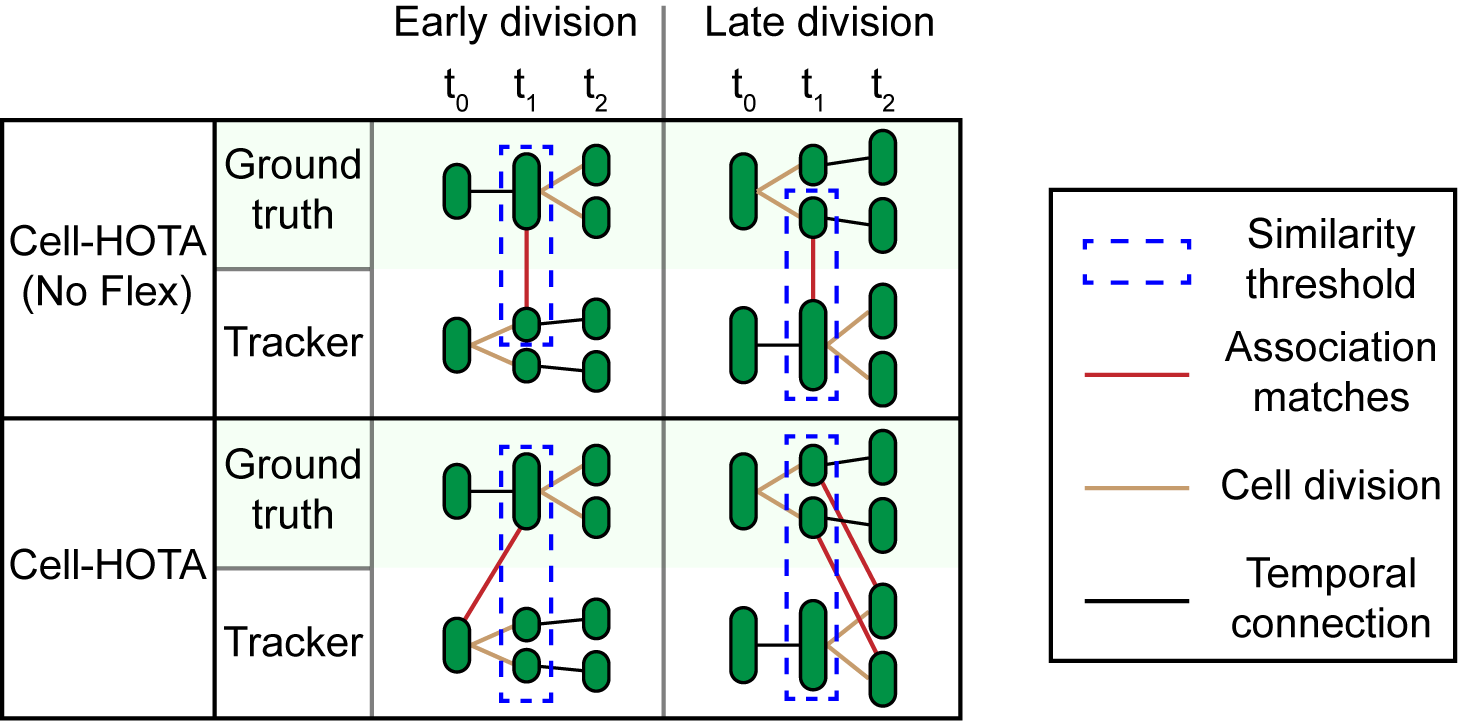

Supplement: S7 Fig — (TIF) [file pcbi.1013071.s011.tif]

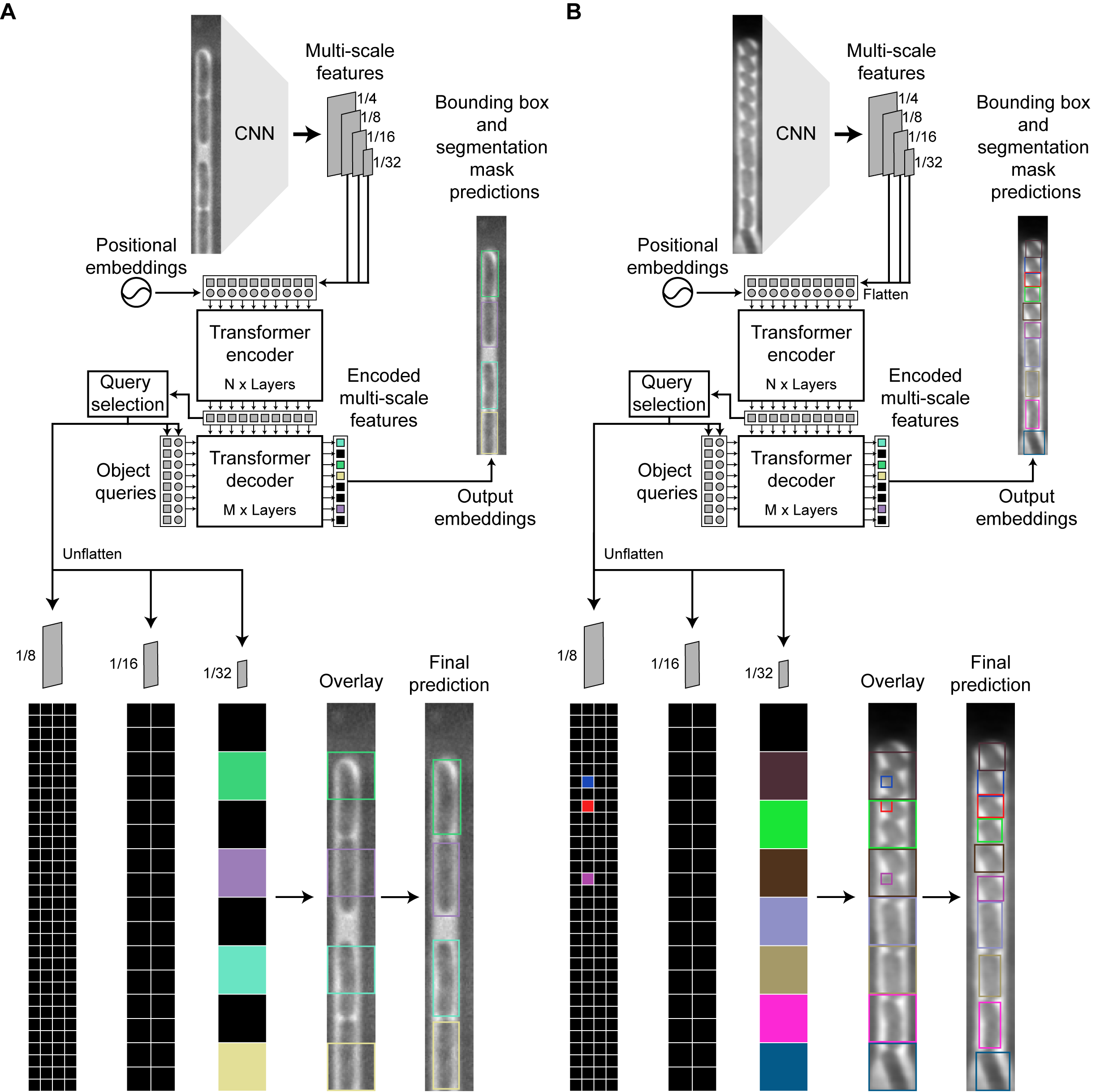

Supplement: S8 Fig — (TIF) [file pcbi.1013071.s012.tif]

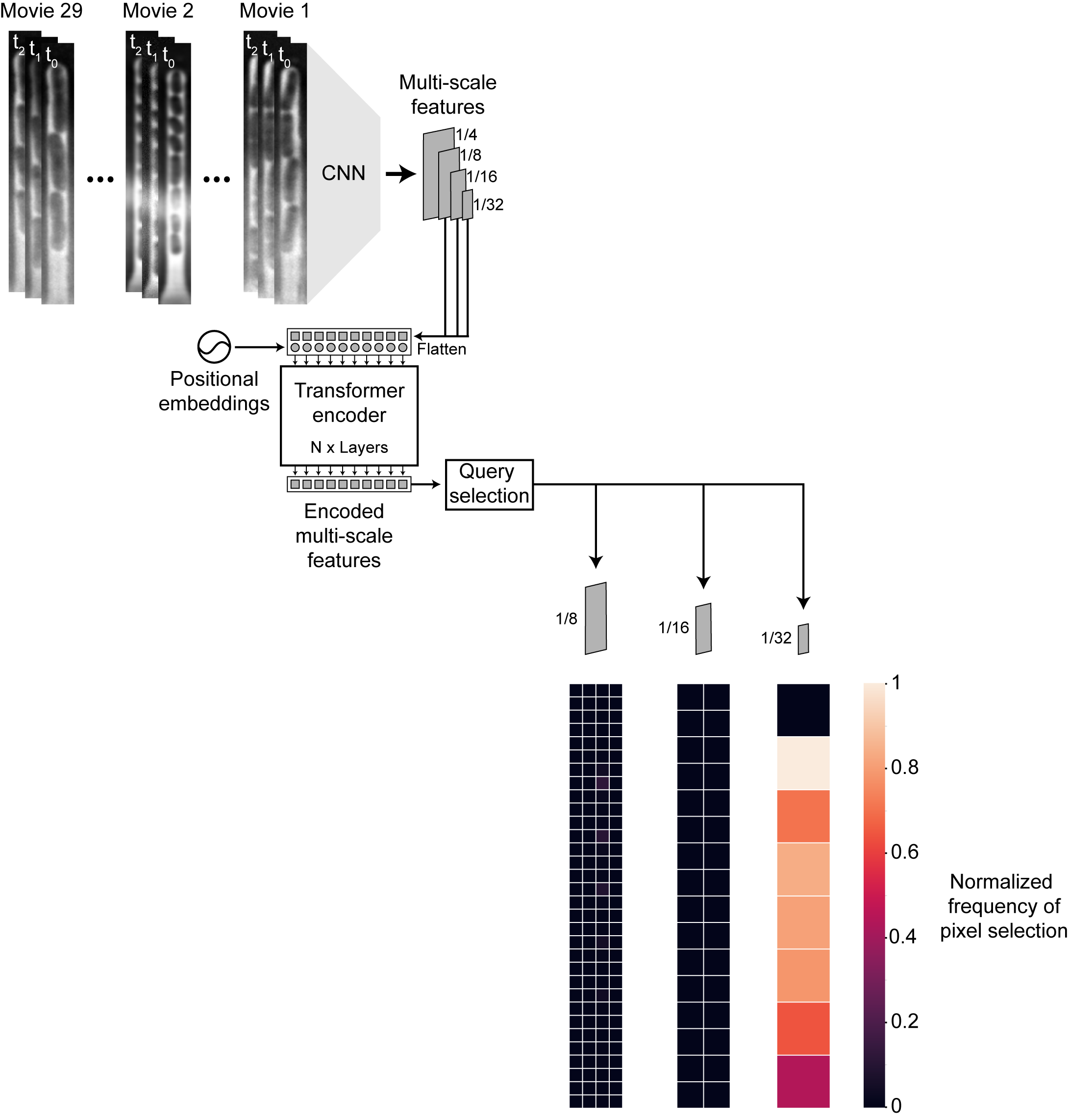

Supplement: S9 Fig — (TIF) [file pcbi.1013071.s013.tif]

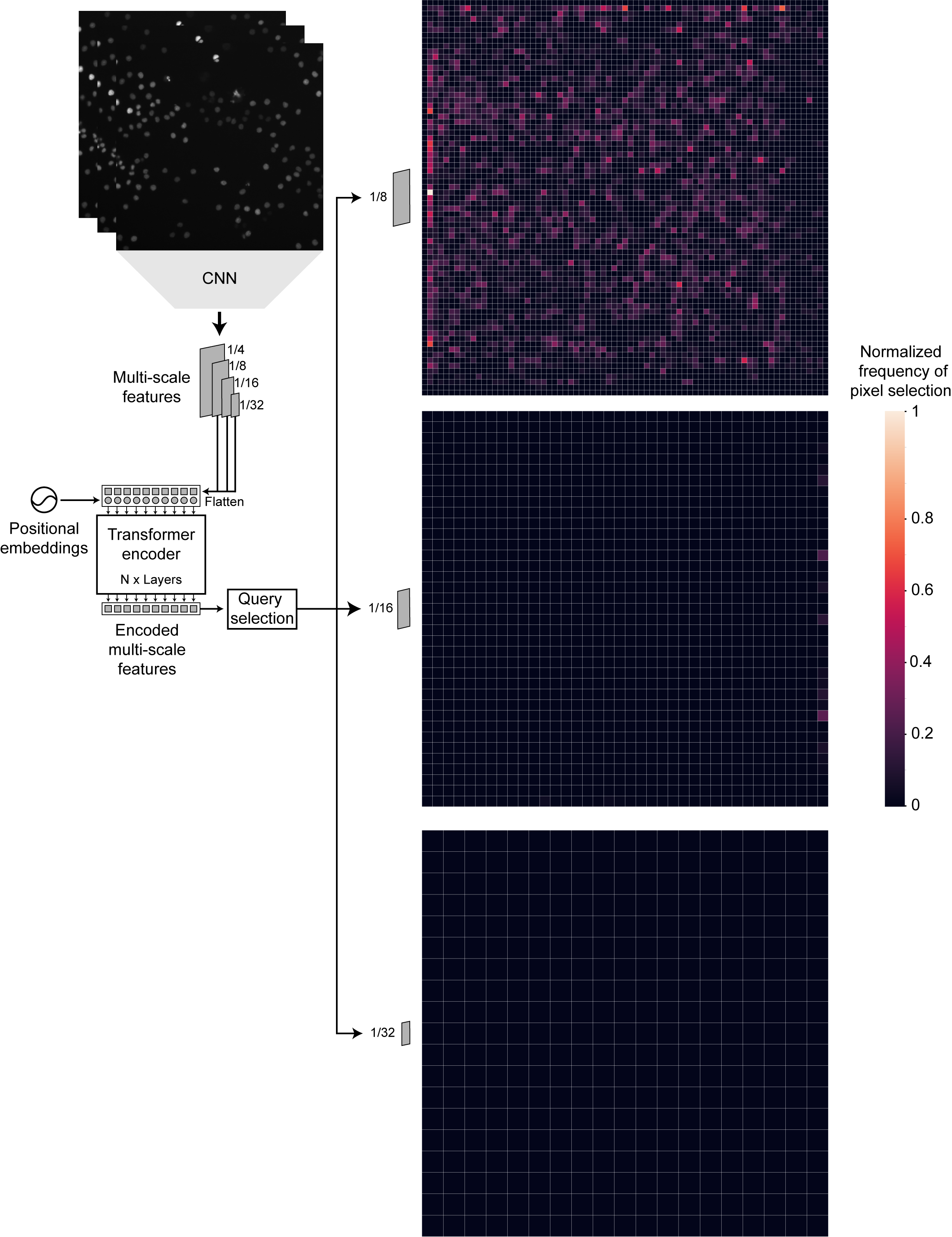

Supplement: S10 Fig — (TIF) [file pcbi.1013071.s014.tif]

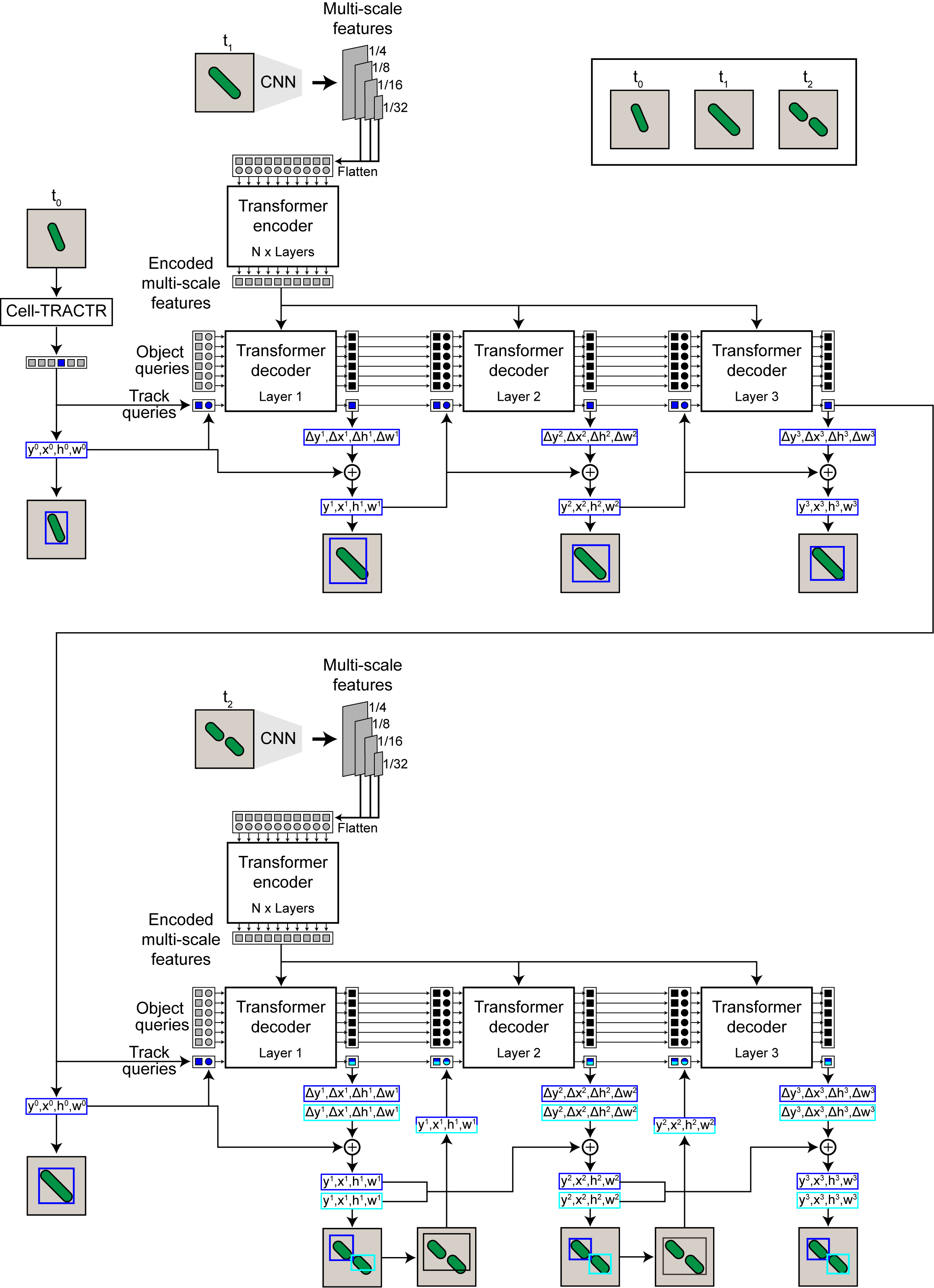

Supplement: S11 Fig — (TIF) [file pcbi.1013071.s015.tif]

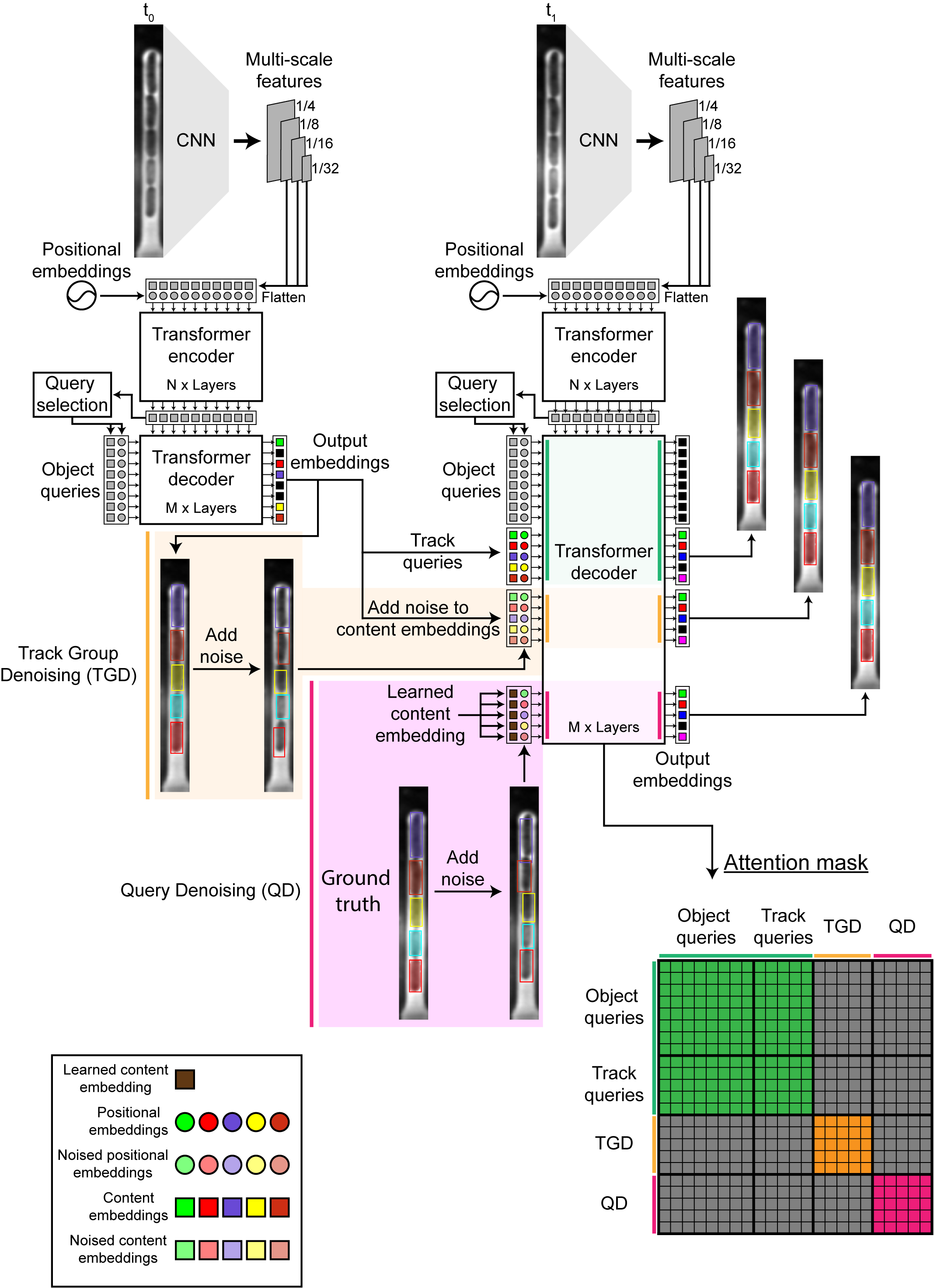

Supplement: S12 Fig — (TIF) [file pcbi.1013071.s016.tif]

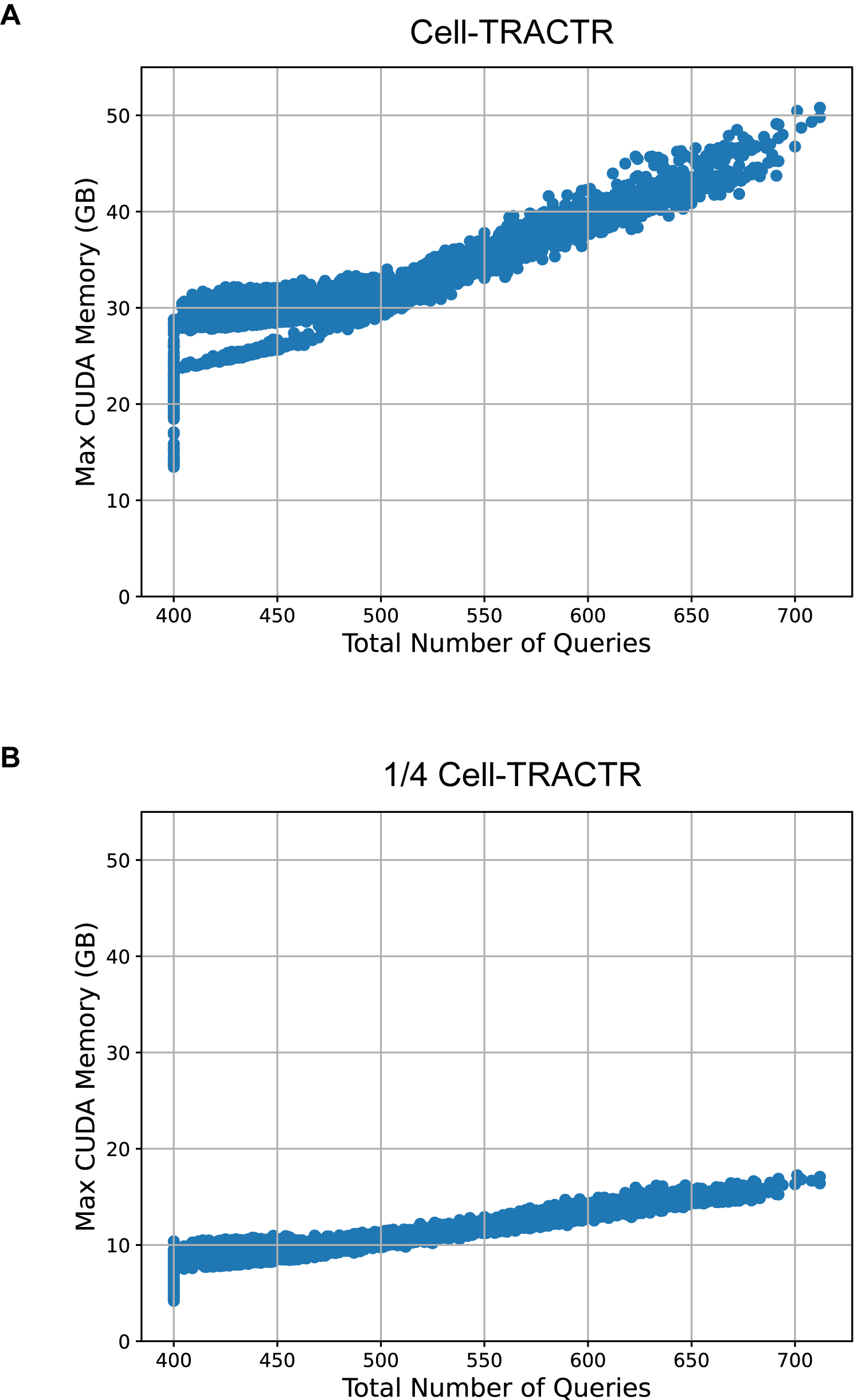

Supplement: S13 Fig — (TIF) [file pcbi.1013071.s017.tif]
