# Supplementary material for: Cell-TRACTR: A transformer-based model for end-to-end segmentation and tracking of cells
Source: PLoS Comput Biol. 2025 May 23;21(5):e1013071. doi: 10.1371/journal.pcbi.1013071 (PMC12101859; doi:10.1371/journal.pcbi.1013071)
Supplement: Attachment — Submitted filename: Response to Reviewers.pdf [file pcbi.1013071.s020.pdf]

Point-by-Point Response to Reviewers
Manuscript Number: PCOMPBIOL-D-24-01171

We appreciate the reviewers' comments and suggestions for improving the content and clarity of the manuscript. We have addressed their concerns in the revised manuscript. A complete list of changes and detailed responses to the comments follows.

Major changes:

- We benchmark against Trackastra and Caliban models.
- We clearly note Cell-TRACTR's performance relative to the state-of-the-art and discuss present limitations and directions for future optimization.
- We include new data and discussion on memory usage.

Reviewer #1

Cell-TRACTR is a transformer-based deep learning algorithm that combines a convolutional neural network for visual feature extraction with a transformer encoder-decoder architecture, inspired by the DETR model from Carion et al. (2020). This method is the first to apply DETR to biological image tracking, but it has some limitations:

Major points

1. Limited Improvement: in comparison to the non-transformer-based methods, the improvement by Cell-TRACTR needs more investigation. The presented method shows little improvement with the first dataset and inconsistent improvement with the second dataset. Especially with the second dataset, the real performance gain is in the cell division accuracy component (DivA) of Cell-HOTA. The reason for this is not clear. This does not necessarily mean that the presented method is ineffective, but it will be more useful for the audience if the authors present more specific use cases where the DivA component would make a crucial difference. Adding such datasets or more detailed analysis on the DeepCell Dataset, such as ablation studies, might prove helpful.

> Thank you for bringing up this concern. Our main goal with this new model is to introduce a novel transformer-based approach that can simultaneously perform cell segmentation and tracking without the need for any post-processing. Thus, our goal was to achieve performance comparable to the current state-of-the-art, and not necessarily to exceed this. We have updated text through the manuscript to clarify this point.
>
> → See Abstract, Results, and Discussion
>
> As described below, we now also benchmark against two additional algorithms to give a clearer view of Cell-TRACTR's performance relative to other high-performing algorithms. Through the sub-metrics $AssA_{0.5}$ and $DivA_{0.5}$, we demonstrate that Cell-TRACTR exhibits strong performance in tracking and division predictions, while performing reasonably on segmentation.
>
> → See Figure 4, Figure 5, discussion throughout the text

2. Scalability Concerns: Due to DETR's bipartite matching, the number of detectable/trackable objects is limited by the specified number of queries (class label, bounding box, and segmentation mask). How does Cell-TRACTR handle images with numerous objects or significant variance in object numbers between samples? Additionally, insights into how computational cost scales with the expected maximum number of objects would be valuable.

> Cell-TRACTR can accurately perform tracking with many cells present, but is also capable of performing well when there are small numbers of cells. For example, the DeepCell dataset contain images that vary from 3 cells to up to 281 cells. We now address this point in the text.
>
> → See Results section "Analyzing mammalian DeepCell microscopy movies"
>
> To address the question about how computation cost, we have added a section in the Supplementary Methods and a new supplementary figure showing how computational cost scales with the total number of queries.
>
> → See Supplementary Methods, Figure S13

Minor points

1. Lack of Comparison with Transformer-Based Models: The method is not compared to other transformer-based models. It would be useful to evaluate its performance against models like Trackastra, followed by cell-segmentation methods, to assess the impact of the transformer architecture.

> Thank you for the suggestion. To address this point, we now evaluate the performance of Trackastra on the bacterial mother machine and DeepCell dataset.
>
> → See Figure 4, Figure 5, and related discussion
>
> For cell-segmentation methods, we acknowledge that the precision of the segmentation head of Cell-TRACTR performs reasonably, but below other algorithms we tested. To help address this point, we reference a new cell segmentation model in the discussion, called CelloType, that demonstrates superior performance compared to other state-of-the-art cell segmentation models. CelloType is based on the architecture of Mask DINO (a DETR-based model) which heavily influenced the segmentation head of Cell-TRACTR, and could be incorporated in the future.
>
> → See Discussion
>
> We also note that Trackastra is specific to cell tracking, and we discuss how its performance is dependent upon segmentation mask quality.

→ See Results sections “Benchmarking Cell-TRACTR and other algorithms” and “Analyzing bacterial mother machine movies”

2. Comparison with Common Workflows: Comparing Cell-TRACTR with popular workflows, such as commonly used configurations from Trackmate software, would be beneficial for biologists.

While TrackMate uses state-of-the-art cell segmentation software, its cell tracking is not currently competitive with other state-of-the-art cell tracking software. Therefore, we decided to exclude it from our analysis. We felt it was more appropriate to compare to DeLTA, EmbedTrack, Trackastra, and Caliban, which are state-of-the-art in cell tracking. However, the point about the benefit of user-friendly workflows is very important and we note this as a future direction for Cell-TRACTR development.

→ See Discussion

3. Figure 2 could be more concise by reducing the number of repetitive objects.

We considered various ways to streamline this figure, but when we made variations and presented them to other researchers, they ultimately preferred the more complete version. Although more detailed, it avoids confusion that arose when parts were omitted. Because of this, we have elected to leave the figure as is in the interest of representing the algorithm accurately.

Reviewer #2

The authors present a deep learning based framework for segmentation and tracking of cells, Cell-TRACTR. Their framework builds upon recent advances in multi-object tracking using end-to-end transformer based architectures and extends these by the ability to handle splitting objects, a feature specifically useful for tracking cells in biological microscopy time-lapses. As a result, the presented framework differs from many existing approaches in that it handles the segmentation and tracking tasks jointly, instead of performing these independently. Specifically, it detects and links cells sequentially across time frames in an end-to-end fashion, avoiding the requirement for significant post-processing steps or pre-engineered features.

The authors evaluate the performance of their framework on two datasets and compare the results to two CNN-based frameworks, one of them being part of their previous work and another one which shares the joint segmentation and tracking approach and performs highly competitively in the Cell Tracking Challenge (CTC). Using the commonly used CTC Cell Tracking Benchmark metric, Cell-TRACTR achieves similar scores to the reference frameworks in the tracking submetric, while exhibiting lower scores in the segmentation submetric and leading to somewhat lower overall performance in this metric.

However, the authors convincingly argue that the CTC metric has shortcomings especially in terms of measuring tracking accuracy and a method's performance in detecting cell divisions. To address these issues, the authors present a new metric, Cell-HOTA, which extends a recently developed and broadly adopted multi-object tracking metric by an additional submetric evaluating the performance in cell division detection. Using this metric, Cell-TRACTR overall performs better than the methods it's compared to, with the division submetric notably being the strongest driver of the comparative advantage.

In my opinion, the presented Cell-TRACTR framework represents a highly interesting contribution to the field by presenting a novel approach to cell segmentation and tracking. The manuscript further provides proof that this end-to-end transformer based method for jointly segmenting and tracking, with simultaneous detection of cell divisions, can yield promising results on microscopy datasets of relevant sizes. Furthermore, in my opinion the presented metric for evaluating cell segmentation and tracking performance, including a detailed discussion on metric properties and limitations, provides another valuable contribution to the field. Furthermore from my point of view the manuscript is well written and I appreciate the detailed introduction and discussion sections, which include a survey of deep learning based methods for segmentation and tracking and position the manuscript within relevant literature.

In the following I'm listing points which are meant as constructive feedback and I believe when addressed by the authors would improve the quality and clarity of the manuscript.

Thank you for your interest and appreciation of our work.

Manuscript

- One of the key aspects of the presented tracking framework is that it builds upon a transformer architecture, which is also mentioned in the title. Accordingly, in the abstract the authors state that

long-range connections are "critical for robust cell tracking". In the introduction, it's mentioned that long-range connections present in transformers and not CNN can be helpful in dealing with substantial shifts in the field of view or highly motile cells. However, I have not found that these statements are backed up quantitatively in the results section. Can these statements be justified quantitatively? E.g. by comparing evaluation metrics of CELL-TRACTR and the CNN-based frameworks on relevant subsets of the example datasets. Alternatively, the datasets could be artificially augmented such that e.g. large shifts would be present and the comparison between the different frameworks could underline the above mentioned statements. I believe that addressing this point is an opportunity to strengthen the results of the manuscript. Otherwise if this is not doable with reasonable efforts, I think the statements involving the long-range properties of transformers should be either clearly attributed to external references in the context of cell tracking or modified/moved to the discussion such that the reader doesn't assume the statements have been specifically shown, which in my opinion is currently the case (i.e. due to presence in abstract and introduction without citation).

> We agree that analyzing large shifts would be very interesting, but given time constraints and requests across the three reviewers, we ended up focusing instead on benchmarking against other algorithms which took substantial effort in some cases. However, to avoid implying results that are not present, we have modified the statements about long-range properties that appeared early in the manuscript and moved these points to the Discussion.
>
> → See Abstract, Introduction, Discussion

- In the discussion, the authors state that "...allow for segmentation and tracking to be accomplished in one unified step which is important for ensuring high accuracy". Is this evidenced in the results section in the sense that jointly performing the tasks increases performance? If not, addressing this could make a strong point. Otherwise, or if this is not feasible, I think it would make sense to clarify this e.g. along the lines of "we assume that one unified step is important for ensuring high accuracy".

> We have added two references backing up this claim that have conducted comprehensive assessments on this point. We have taken care to avoid language implying that we tested this directly in our case.
>
> → See Discussion

- In the discussion, the authors state "We found that Cell-TRACTR excelled in association and division accuracy while all three models demonstrated comparable detection capabilities." and "We found that Cell-TRACTR delivered strong performance on both a bacterial mother machine dataset and a mammalian dataset from DeepCell". As mentioned above, the segmentation/detection submetrics in many evaluations (particularly the overall ones) are lower for CELL-TRACTR than for the comparison frameworks. Unless I misinterpreted the metrics, I think mentioning and interpreting this in the discussion would be important. Further if there are specific limitations of CELL-TRACTR with respect to segmentation / detection the authors are aware of, it would be useful to mention here.

We have modified the text to make it clear that Cell-TRACTR did not perform as well on the main Cell-HOTA metrics due to reduced segmentation precision. Throughout the manuscript we have also clarified wording to avoid any incorrect implications about Cell-TRACTR's performance.

→ See Figure 4, Figure 5, and associated discussion

- In the results the authors state "This end-to-end model, where the input is the raw image and the output is embeddings that represent the position and lineage information for each cell, eliminates the need for post-processing techniques like non-maximum suppression that are required for other tracking by detection approaches." If I understood correctly, what is end-to-end is the segmentation and tracking (or "linking" in this case) between consecutive timeframes, however obtaining position and lineage information for a full timelapse requires following the cell identities over the time frames and across cell divisions. If this is correct, given the centrality of the 'end-to-end' property of the framework, I think the above statement should be clarified such that it's more explicit to the reader which part of the framework works in an end-to-end fashion.

We have clarified this statement by adding more detail.

→ See Results section "Cell-TRACTR simultaneously segments and tracks cells"

- While visually very clear and pleasing as figures in general, in my opinion figure 1 is slightly confusing regarding the model schematic. While the figure legend titles "Overview of Cell-TRACTR analyzing time-lapse microscopy movies", panel C only depicts the detection layers of the model. In my opinion, this could either be stated more clearly in the legend, or a more complete schematic of the model could be included here which would fulfill the function of a more complete overview, potentially even depicting the cell division handling mechanism.

We have updated the figure legend title. We chose not to include tracking in the model in panel C for the sake of clarity, instead leaving it as the focus of Figure 2 (Reviewer #1 already noted concern about Figure 2 being too detailed, so we were hesitant to combine these).

→ See Figure 1 caption

- The authors mention that training Cell-TRACTR in its current form takes long (e.g. more than a week for the DeepCell dataset), but can run into out-of-memory issues. Can the authors make an approximate statement regarding how much memory is required to train Cell-TRACTR as a function of dataset size and relevant hyperparameters?

We added a new supplementary figure and a section in the Supplementary Methods to address this point.

→ See Figure S13, Supplementary Methods

- Can the authors include statements about which type of datasets the framework is expected to work specifically well / less well on? E.g. regarding crowding / cell motility / As an idea for this, the manuscript mentions that the DeepCell dataset is large and diverse. This could be leveraged to pinpoint strengths and weaknesses quantitatively.

We have added this in Discussion.

→ See Discussion

Software

- When following the installation and usage instructions in the Cell-TRACTR repository, I stumbled upon a few problems:
  - The last line in the installation instructions aiming to install the MultiScaleDeformableAttention package points to a file in the repo which doesn't exist (src/Cell-TRACTR/models/ops/setup.py)

Thanks for pointing this out. There was a typo in the path, which we have now updated in the README.md.

  - After installing MultiScaleDeformableAttention from different sources, the following packages are required but not installed:
    - python-ffmpeg
    - scikit-image
    - fvcore

Thank you for bringing this to our attention. We have updated the Installation instructions to include these packages.

  - I could not start training after approx. an hour of debugging. I'm stuck at `python \-i Cell-TRACTR/src/train.py with dataset='moma'` which gives the error: `TypeError: ms\_deform\_attn\_forward(): incompatible function arguments.`

We have updated how multi-scale deformable attention is installed, which may resolve this issue. Unfortunately, this appears to be a common problem with deformable attention (according to GitHub issues, e.g. https://github.com/fundamentalvision/Deformable-DETR/issues/220) where many people have run into similar problems. Hopefully our updates have helped to address this for Cell-TRACTR, though it is difficult to diagnose this specific error without further details.

- While the above might be a list of encountered problems that could have been avoided, in my opinion this highlights that it's not completely straight-forward to reuse the code. Given that the framework is meant to be used by the community, I believe that making the software more available would be very valuable, increase its adoption and strengthen its overall impact. Specifically, it could be very helpful if the authors provided a docker container containing a functional installation of the code repository. Alternatively, if this is not feasible, the authors could improve the installation instructions and make sure they work on several independent machines.

We have improved and updated the installation clear. We also refer users to the Deformable DETR GitHub page, which could be a useful source for installation issues.

→ See the Cell-TRACTR repository on GitLab (https://gitlab.com/dunloplab/Cell-TRACTR)

- The code repository for Cell-HOTA indicates that code was tested on Python 3.7. Given that this python version is no longer widely supported, confirming functioning code with Python 3.10 would be important in my opinion.

We have updated Cell-HOTA to be compatible with Python 3.10. This has been updated in the README.md

Reviewer #3

Major comments:

1. It's difficult to assess the proposed method's performance in relation to the existing literature as a whole because it was evaluated in only two datasets against two algorithms (one of which is from the same authors). This is especially true since they propose a new metric, which is indeed needed. However, it reduces the comparability to existing work despite them reporting the CTC score, which, as they said, is problematic. We recommend comparing it with CALIBAN [22], which produced the DeepCell dataset used in this paper, and Trackastra [40], which is also a transformer-based method for tracking and the current state-of-the-art in the DeepCell dataset. Both of these methods have their code and weights publicly available.

Thank you for the suggestion. We now evaluate Caliban and Trackastra on the DeepCell dataset. In addition, we compare the performance of Trackastra on the bacterial mother machine dataset.

→ See Figure 4, Figure 5, and associated text

2. A concurrent work, Cell-specific Higher Order Tracking Accuracy (CHOTA)[1*, 2*], which extends HOTA for cell tracking, was recently proposed. It doesn't seem to be the same metric as yours, so it would be interesting to highlight the differences between the two from the theoretical and experimental point of view (e.g., how they measure different things).

This pre-print appeared while the manuscript was under review, and we agree that this comparison is very important and relevant. We now discuss similarities and differences between the two approaches in the Discussion. Both CHOTA and Cell-HOTA offer distinct advantages over the original CTC score.

→ See Discussion

3. It's not clear how to obtain the network weights used in the experiments. The weights are as important as making the code available, especially in this case where server-grade GPU (V100/A100) are needed to train the models. Otherwise, the reproducibility and application of the proposed method are greatly reduced.

We have resolved this by posting the weights to Zenodo.

→ See Code Availability and Datasets sections

4. This method's application seems a bit limited because it requires an extensive amount of GPU RAM, an A100 for 584x600 pixel images with a batch size of 1, which are small images for today's standards. Plus, it seems to require a considerable amount of training data. How does it perform in videos from the Cell Tracking Challenge which are often more challenging than the DeepCell dataset?

We have added a section on the current computational constraints of Cell-TRACTR in the Supplementary Methods and a new supplementary figure addressing this.

→ See Supplementary Methods, Figure S13

The CTC datasets are very small and often partially annotated and are thus not ideal for training. Instead, we focus on the larger, fully annotated bacterial mother machine and DeepCell datasets. We note this in the manuscript.

→ See Introduction

Minor comments:

5. Omnipose appears twice in the references [8, 32]

Fixed.

6. From section "Training EmbedTrack." How are (horizontal or vertical) flip augmentations incompatible with a fixed crop size?

We have updated the text to fix this issue. EmbedTrack used a data augmentation function that would randomly rotate an image 90 degrees, which is incompatible with non-square crops, therefore we did not include it during training.

→ See Methods section “Training EmbedTrack”

[1*] Kaiser, Timo, Vladimir Ulman, and Bodo Rosenhahn. "CHOTA: A Higher Order Accuracy Metric for Cell Tracking." arXiv preprint arXiv:2408.11571 (2024).
[2*] http://www.bioimagecomputing.com/program/selected-contributions/
