# Supplementary material for: Cell-TRACTR: A transformer-based model for end-to-end segmentation and tracking of cells
Source: PLoS Comput Biol. 2025 May 23;21(5):e1013071. doi: 10.1371/journal.pcbi.1013071 (PMC12101859; doi:10.1371/journal.pcbi.1013071)
Supplement: Attachment — Submitted filename: Response_to_Reviewers_auresp_2.pdf [file pcbi.1013071.s021.pdf]

Point-by-Point Response to Reviewers
Manuscript Number: PCOMPBIOL-D-24-01171

Reviewer #1
The authors have thoroughly addressed all of my concerns and those of the other reviewers.

Thank you for the positive comments.

Reviewer #2
My concerns regarding the manuscript have been addressed by the authors.

Also, following the improvements in installation instructions and the code, I've been able to successfully train and apply Cell-TRACTR on the "moma" dataset, after fixing two (minor) code problems:

First, during training the following error occurred:
```
File ".../Cell-TRACTR/src/trackformer/datasets/mot.py", line 101, in __getitem__
man_track_id = self.coco.imgs[idx]['man_track_id']
KeyError: 'man_track_id'
```

I could fix this by adding the following line in the script which transforms datasets from CTC to COCO format, scripts/create_coco_dataset_from_CTC.py, after line 98 (relative to commit 32d37554b2cb9b83b271d5ea0f163f6b29250df6):
`'man_track_id': f'{dataset_name}',

Second, also during training there was a problem with the `datapath` in src/pipeline.py, which I could fix by changing line 127 from

`datapath = args.data_dir / 'CTC_datasets' / dataset / 'CTC' / 'test'`
to
`datapath = args.data_dir / dataset / 'CTC' / 'test'`

Given that all of my points have been addressed by authors and the code problems above are minor and easily fixed, I recommend the manuscript for publication.

We appreciate your recommendation that the manuscript is suitable for publication. Thank you for pointing out the issue that you encountered during training. We have updated the code to fix this.

→ Code updates on Gitlab

Reviewer #3

While the authors have addressed some concerns raised during the previous review, key issues remain unresolved, and the additional experiments fail to strengthen the manuscript's contributions.

Major comments:

- Computational Inefficiency and Resource Intensity: Despite the added discussion on computational constraints, the reliance on an A100 GPU for training for images with the size of 584x600, which is not that large in the broad scope of microscopy. Thus, the method is impractical for widespread adoption unless they show their pre-trained weights can be generalized to other datasets.

> This is a valid point. The transformer architecture brings many benefits for cell segmentation and tracking, but at the cost of computational complexity. This is a known limitation and there are many current efforts aimed at improving efficiency. For example, for the DETR-based architecture we use, there are numerous new studies that introduce modified architectures to address this point, and we have cited several examples of these works in the Discussion, including new results that have appeared while this paper has been under review.
>
> However, the reviewer's point is that the Cell-TRACTR model is not practical for widespread adoption in its current form. We think it is worth making several counterpoints. First, even in its present form the algorithm is very appropriate for analysis of microfluidic experimental data like those derived from the mother machine. In itself, this is a significant contribution as use of these devices is widespread. Indeed, many papers focus on developing methods that work for the mother machine device alone (a recent example is Thiermann *et al*. 10.7554/eLife.88463). We see the algorithm's ability to both handle mother machine data and to show strong potential to extend to two dimensional analysis of different cell types as a bonus. Second, as we note above and describe in the Discussion, there are active efforts to improve the computational efficiency of DETR-based models. Incorporating these advances is likely to improve the scalability of the model, but we feel is outside the scope of the present work.
>
> In response to this comment, we did attempt to show that the pre-trained weights could be generalized to other datasets. While we were able to train a generalizable cell segmentation model, like the DETR-based CelloType model, transferring this knowledge to cell tracking was more challenging. It remains unclear whether components, such as the backbone and encoder, should be frozen or fine-tuned (e.g. with a reduced learning rate), particularly given the added complexity of modeling cell division. Ultimately, we concluded that further work is needed to understand how to best apply transfer learning. Because our results in this direction were not concrete, we have not included them in the present manuscript, but we do now describe this as a potential future direction in the Discussion.
>
> → See Discussion

Performance on DeepCell: The results on the DeepCell dataset do not favor the claims that this method's performance is comparable with current approaches such as Trackastra and Caliban. While it could be argued that it works on the Mother Machine dataset, it's an incredibly limited scenario of 1D tracking because the cells' movement is mostly vertical because of the Mother Machine device.

We have shown that performance of Cell-TRACTR algorithm is on par with Caliban for the widely-used similarity threshold $\alpha = 0.5$ (Fig. 5B). Although performance degrades slightly with higher values of α, Cell-TRACTR still continues to outperform Trackastra, DeLTA, and EmbedTrack (Fig. 5A). It is only at the most stringent α values where this relationship changes, but these correspond to unlikely use cases where performance on cell segmentation must be near perfect. Although Caliban does outperform Cell-TRACTR slightly, this is not a surprising result because Caliban was specifically designed and customized for the DeepCell dataset. Indeed, it is a sign of strong performance that Cell-TRACTR performs close to as well for many values of α. The reviewer also mentions the performance of Trackastra. We note that Trackastra only performs tracking and not cell segmentation. We used the cell segmentation data from Caliban to give it the best shot at performing well and included this algorithm because it had been requested in the prior round of review. But it is worth remembering that Trackastra only performs a portion of what the other algorithms accomplish.

- Unaddressed Reviewer Comments: Reviewer 2's suggestions for experiments corroborating the authors' original claims were mainly addressed by tuning down their contributions. While this makes the paper more factual, it highlights how the method's performance could be improved.

We acknowledge this point, and noted these as future directions in the prior round of revision.

In summary, the authors have made efforts to address the reviewers' feedback. However, the limitations of the method persist, and the application of this method beyond the experiments shown here is questionable due to the availability of more computationally efficient and accurate methods.
